# Gastrodin alleviates premature senescence of vascular endothelial cells by enhancing the Nrf2/HO‐1 signalling pathway

**DOI:** 10.1111/jcmm.18089

**Published:** 2023-12-25

**Authors:** Pengfei Tong, Ke Tian, Jiajia Bi, Ruihua Wang, Zhengfeng Wang

**Affiliations:** ^1^ Neurosurgery Department The Third People’s Hospital of Henan Province, Zhongyuan District Zhengzhou City Henan Province China; ^2^ Nuclear Medicine Department The First Affiliated Hospital of Zhengzhou University, Erqi District Zhengzhou City Henan Province China; ^3^ Neurosurgery Department The First Affiliated Hospital of Zhengzhou University， Erqi District Zhengzhou City Henan Province China

**Keywords:** cell senescence, cerebrovascular disease, gastrodin, Nrf2/HO‐1, oxidative stress, proliferation

## Abstract

Endothelial dysfunction is an independent risk factor for stroke. The dysfunction of endothelial cells (EC) is closely concerned with EC senescence. Gastrodin (GAS) is an organic compound extracted from the dried root mass of the Orchidaceae plant Gastrodiae gastrodiae. It is used clinically to treat diseases such as vertebrobasilar insufficiency, vestibular neuronitis and vertigo. In the present study, we used hydrogen peroxide (H_2_O_2_)‐induced human umbilical vein endothelial cells (HUVECs) to establish an in vitro EC senescence model and to investigate the role and mechanism of GAS in EC senescence. It's found that H_2_O_2_‐treated HUVECs increased the proportion of senescence‐associated β‐galactosidase (SA β‐gal) positive cells and the relative protein expression levels of senescence‐associated cyclin p16 and p21. In addition, GAS reduced the proportion of SA β‐gal positive cells and the relative protein expression levels of p16 and p21, and increased the proliferation and migration ability of HUVECs. Meanwhile, GAS increased the expression of the anti‐oxidative stress protein HO‐1 and its nuclear expression level of Nrf2. The anti‐senescence effect of GAS was blocked when HO‐1 expression was inhibited by SnPPIX. Furthermore, absence of HO‐1 abolished the effect of GAS on HUVEC proliferation and migration. In conclusion, GAS ameliorated H_2_O_2_‐induced cellular senescence and enhanced cell proliferation and migration by enhancing Nrf2/HO‐1 signalling in HUVECs. These findings of our study expanded the understanding of GAS pharmacology and suggested that GAS may offer a potential therapeutic agent for stroke.

## 1 INTRODUCTION

Cerebrovascular disease is one of the leading causes of death and disability worldwide.^1^ It is the first cause of death and the leading cause of adult disability in China.^2^ Vascular endothelial cells (EC) play an important role in maintaining vascular homeostasis, such as maintaining blood flow, vascular tension, responding to hormones, neurotransmitters and physiological stimuli, and secreting factors related to vascular regulation.^3^ A growing body of research suggests that vascular EC dysfunction is critically associated with cerebrovascular disease.^4^, ^5^ Senescent ECs exhibit functional deficits, adopt enlarged and flattened morphologies and lose their ability to replicate.^6^ This compromises the integrity of the endothelial barrier function and facilitates the onset of a range of cerebrovascular diseases, such as intracerebral haemorrhage, cerebral embolism and intracranial aneurysms.^7^, ^8^ Cerebrovascular diseases impose a high social, familial and economic burden.^9^ Therefore, therapeutic prevention or attenuation of EC senescence has important implications for the treatment of cerebrovascular diseases.

Cellular senescence involves complex etiological and pathophysiological processes determined by a multitude of factors, including oxidative stress, inflammation and shorten length of telomeres.^10^, ^11^ It is a state of irreversible growth arrest that cells enter into in response to various stressors or as a result of normal aging processes. It is characterized by a stable and permanent cessation of cell division, accompanied by distinct morphological and molecular changes in the cell^12^; This process acts as a safeguard mechanism to prevent the proliferation of damaged or potentially harmful cells, such as those with extensive DNA damage or oncogenic mutations. By halting the division of these cells, cellular senescence helps to maintain genomic stability and suppress the development of cancer.^13^ In vascular ECs and other cell types, characteristic indicators of senescence include increased expression of senescence‐associated β‐galactosidase (SA β‐gal), p16 and p21, and adoption of a senescence‐associated secretory phenotype (SASP).^8^, ^14^ Oxidation–reduction (redox) reactions contribute to fundamental physiological processes, regulate various aspects of cellular activity and maintain cellular and organismal homeostasis. When the intracellular redox reaction is broken, mitochondrial function is impaired and a large amount of ROS is produced. Excessive ROS cannot be removed in time, damaging the DNA integrity and the length of telomerase in cells and triggering the senescence‐related process.^12^ Ultimately, these senescence‐inducing signals will trigger the p53/p21 and p16/ retinoblastoma protein tumour suppressor pathways as final effectors of the aging program.^15^ Therefore, disruption of the redox reactions homeostasis can lead to cell dysfunction and death.^11^, ^16^

Heme oxygenase‐1(HO‐1) is a pivotal stress‐inducible enzyme that protects cells from oxidative injury by catalysing the conversion of heme into biliverdin, carbon monoxide and iron ions.^17^ HO‐1 expression is induced in most cell types upon oxidative and inflammatory stimuli.^18^, ^19^ Nuclear factor erythroid 2‐related factor 2 (Nrf2) is a transcription factor that acts as a master regulator of the expression of HO‐1 and other antioxidant genes.^20^, ^21^ When cells are exposed to oxidative stress, Nrf2 dissociates from the cytosolic repressor protein Kelch‐like ECH‐associated protein 1 (Keap1).^22^ Subsequently, Nrf2 is translocated into the nucleus, where it binds DNA sequences known as antioxidant response elements (ARE) to regulate the expression of various downstream genes, promoting the restoration of redox homeostasis.^20^, ^23^ Therefore, we speculated that Nrf2/HO‐1 signalling pathway activation exerts an essential role in protecting ECs from cell senescence induced by oxidative stress.

Based on an increasing number of studies characterizing the bioactive ingredients and pharmacological properties of herbal extracts and polyherbal formulations used in traditional Chinese medicine (TCM), the therapeutic potential of TCM for prevention and treatment of cardiovascular and cerebrovascular diseases has attracted considerable attention in recent years.^24^ Gastrodin (GAS), a phenolic glycoside, is the main active compound of a Chinese herbal medicine obtained from the roots of rhizoma Gastrodiae, and is also produced by chemical synthesis. GAS has been employed clinically as a TCM therapy for vascular and neurological conditions such as hypertension, cerebral ischemia and epilepsy.^25^ Zhang et al. reported that GAS exposure alleviates H_2_O_2_‐induced injury in mouse liver sinusoidal ECs through p38 MAPK phosphorylation and activation of Nrf2 signalling.^26^ More recently, GAS treatment was shown to alleviate acetaminophen‐induced liver injury in mice through inhibition of MAPK and stimulation of Nrf2‐dependent pathways.^27^ However, whether GAS exerts anti‐senescence effects on vascular ECs remains unknown. Thus, in this work we characterized the effects of GAS on the expression of senescence markers, the proliferation potential and the migratory capacity of cultured ECs challenged with H_2_O_2_, and investigated also the underlying molecular mechanisms.

## 2 MATERIALS AND METHODS

### 2.1 HUVECs culture and treatment protocols

Human umbilical vein endothelial cells (HUVECs) were purchased from American Type Culture Collection. All cells were cultured in endothelial cell medium (ECM) (ScienCell 1001; USA) supplemented with 5% fetal bovine serum (FBS), 1% endothelial cell growth supplement, 1% penicillin/streptomycin (P/S) at 37°C in 5% CO_2_. To select a suitable concentration of H_2_O_2_ for the HUVEC senescence model, cell viability assays were conducted in cells exposed for 2 h to different concentrations of H_2_O_2_ (0, 50, 100, 200 and 400 μM) in serum‐free medium. GAS (purity >98%) was obtained from Shanghai Yuanye Bio‐Technology Co., Ltd. To assess the effect of GAS on HUVECs viability, different concentrations of GAS (0, 6.25, 12.5, 25, 50,100 and 200 μM) were in complete medium were added to HUVECs for 24 h at 37°C. After determining optimal, non‐cytotoxic concentrations of H_2_O_2_ and GAS, the effects of GAS on senescence markers and on cell proliferation and migration abilities were assessed by incubating HUVECs with GAS (50 or 100 μM) 6 h before and over the 24 h that followed H_2_O_2_ addition. This experiment is grouped as follows: Control group (HUVECs), senescence group (HUVECs exposed to 100 μM H_2_O_2_), 50 μM gastrodin + senescence group, 100 μM gastrodin + senescence group To evaluate the role of HO‐1 in the GAS‐mediated cytoprotection, prior to GAS treatment, HUVECs were pre‐treated with 20 μM tin‐protoporphyrin IX (SnPPIX; MedChemExpress), a specific HO‐1 inhibitor, for 2 h. All experiments were repeated three times.

### 2.2 Cell viability assay

Cell Counting Kit‐8 (CCK‐8; Dojindo Co., Kumamoto, Japan) was used to analyse cell viability according to the manufacturer's instructions. In brief, 8 × 10^3^ HUVECs per well were cultured in 96‐well plates, and incubated with ECM at 37°C with 5% CO_2_ for 24 h. Depending on the experiment, the cells are treated and grouped accordingly. After the cells in each group were treated as required, cells were washed with PBS and 10 μL of CCK‐8 solution plus 90 μL of FBS‐free ECM were added to each well. After 2 h, optical density was measured at 450 nm in a microplate reader (BioTek Instruments, Inc.).

### 2.3 Senescence‐associated β‐galactosidase staining

H_2_O_2_‐induced cellular senescence was detected by using a SA β‐gal staining kit (Beyotime Biotechnology, Shanghai, China), following to the manufacturer's protocol. Briefly, HUVECs were cultured in six‐well plates, treated with or without H_2_O_2_, washed three times with PBS, and fixed with 1 mL of fixative solution 15 min. Each well was then incubated with 1 mL of SA β‐gal staining mixture overnight at 37°C staining‐mixture. The percentage of SA β‐gal ‐positive cells was calculated from images taken on a bright field microscope (TS100; Nikon Corporation) at 100× magnification using ImageJ software (National Institutes of Health, USA). Senescent cells become larger and stained blue. Three fields were randomly selected for each group. Count at least 400 cells in each field and then count the ratio of positive cells to total cells All experiments were repeated for three times.

### 2.4 Cell proliferation assay

Cell proliferation was detected by EdU‐488 staining using a BeyoClick™ EdU Cell Proliferation Kit (Beyotime, Shanghai). In briefly, following experimental treatments, HUVECs were incubated with 10 μM EdU for 2 h. The medium was then removed and the cells washed with PBS. Cells were then fixed in 4% paraformaldehyde at room temperature for 15 min, permeabilized with 0.3% Triton X‐100 for 15 min and incubated with the Click Additive Solution for 30 min in the dark. After, nuclear staining with Hoechst 33342, images were captured by a fluorescence microscope. The percentage of EdU positive cells was calculated by ImageJ software. Three fields were randomly selected for each group. The ratio of the number of proliferating cells (cells stained green) to the total number of cells (cells stained blue) was calculated for each field of view. All experiments were repeated for three times.

### 2.5 Cell migration assay

Cell migration potential was assessed by scratch wound‐healing assays. Briefly, HUVECs were plated in six‐well plates (2 × 10^5^ cells/well; three replicates per treatment) cultured at 37°C for 24 h and treated for 2 h with or without H_2_O_2_. Cells were then left untreated or incubated with 50 or 100 μM GAS until cell confluence was >90%. A sterile 200‐μL pipette tip was used to scratch the cell monolayer cells across the center of the well. The wells were then washed with PBS and fresh culture medium was added. HUVECs were photographed at both baseline (0 h) and after 12 h under a light microscope at 40× magnification, and migration rate was quantitatively measured with ImageJ software using the formula: Wound closure (%) = (A0–A1)/A0 × 100, where A0 and A1 represent the wound area at baseline and after 12 h, respectively.

### 2.6 Western blot analysis

HUVECs were lysed in RIPA buffer with PMSF (New Cell & Molecular Biotech Co., Ltd., Suzhou China) and protein contents measured by the BCA method (Epizyme, Shanghai). Samples were denatured in SDS‐PAGE protein loading buffer by boiling for 10 min at 100°C. Equal amounts of proteins for each sample were separated by SDS‐PAGE and transferred to PVDF membranes by electroblotting. The membranes were blocked with protein‐free rapid blocking buffer (Epizyme) at room temperature for 15 min, washed with TBST three times (5 min/wash), and incubated at 4°C overnight with primary antibodies: anti‐p16‐INK4a (Cat. no. 380963; 1:1000 dilution; ZENBIO, Chengdu, China), anti‐P21 (Cat. no. 381102; 1:1000 dilution; ZENBIO Chengdu, China), anti‐Nrf2 (Cat. no. 380773; 1:1000 dilution; ZENBIO Chengdu, China), anti‐HO‐1 (Cat. no. 380753; 1:1000 dilution; ZENBIO Chengdu, China), anti‐GAPDH (Cat. no. 380626; 1:1000 dilution; ZENBIO Chengdu, China), Histone H3 (Cat. no. 9715s; 1:1000 dilution; CST, USA). All the antibodies were diluted using primary antibody dilution buffer for western blot (Beyotime, Shanghai). Following three washes in TBST, the membranes were incubated with HRP‐conjugated goat anti‐rabbit IgG (Cat. no. A21020; 1:10,000 dilution; Abbkine, Wuhan, China) for 2 h at room temperature. Bands were visualized using ECL detection system reagents (Pierce; Thermo Fisher Scientific, Inc.) and grey values for each band quantified and normalized against GAPDH expression by densitometry using ImageJ software.

### 2.7 Statistical analysis

Data are expressed as the mean ± SD from at least three independent experiments. All statistical analyses were performed by Graph Pad Prism8.0 statistical software. Comparisons among groups were performed by anova and Tukey's test for Paired data. *p* < 0.05 was considered significant.

## 3 RESULTS

### 3.1 Establishment of an in vitro model of vascular EC senescence

**FIGURE 1:**
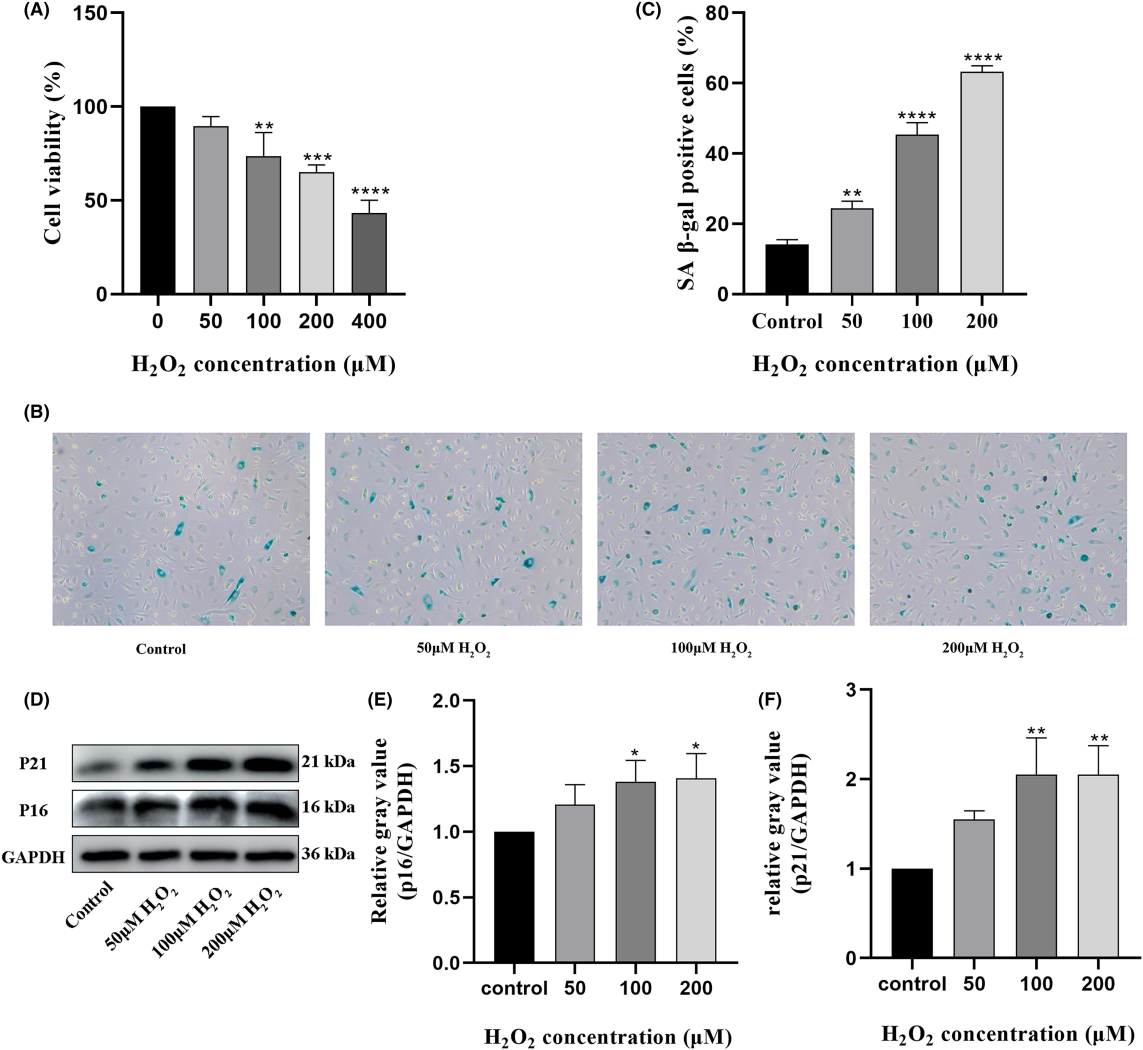
Establishment and characterization of an in vitro HUVECs senescence model. (A) Different concentrations of H_2_O_2_ (0, 50, 100, 200, and 400 e) were applied to cultured HUVECs for 2 h. A CCK‐8 assay was used to evaluate cell viability. (B, C) SA β‐gal staining was performed to evaluate cell senescence. Senescent cells become larger and stained blue (Magnification ×100) (D) Determination of p16 and p21 protein expression by western blotting. (E, F) Densitometric quantification of p16 and p21 protein expression based on western blot assays. *p < 0.05, **p < 0.01, ***p < 0.001, ****p < 0.0001 versus control. CCK‐8, Cell Counting Kit‐8; HUVECs, human umbilical vein endothelial cell; SA β‐gal, senescence‐associated β‐galactosidase.

To establish an in vitro model of H_2_O_2_‐induced senescence in vascular ECs. HUVECs grown to >60% confluence was first incubated with 0, 50, 100, 200 or 400 μM H_2_O_2_ for 2 h. Results of CCK‐8 assays indicated that cell viability was reduced in an H_2_O_2_ dose‐dependent manner, decreasing to below 50% at the 400 μM dose (Figure 1A). Next, the optimal H_2_O_2_ concentration for further experiments was further assessed based on staining of SA β‐gal, a widely used cellular senescence marker, following 24 h incubation after 2 h treatment in 50, 100 or 200 μM H_2_O_2_. As shown in Figure 1B,C, the percentage of SA β‐gal‐positive cells increased in a H_2_O_2_ dose‐dependent manner. Additionally, western blot analysis confirmed that the expression of two cell senescence marker proteins p16 and p21 was also increased after H_2_O_2_ treatment (Figure 1D). As shown in Figure 1E,F, relative p16 and p21 protein expression levels were comparable among cells treated with 100 and 200 μM H_2_O_2_. However, since viability was lower in cells exposed to 200 μM H_2_O_2_, an H_2_O_2_ concentration of 100 μM was selected to establish the HUVEC senescence model for subsequent experiments.

### 3.2 Gastrodin increases the viability in H_2_O_2_ ‐treated HUVECs

**FIGURE 2:**
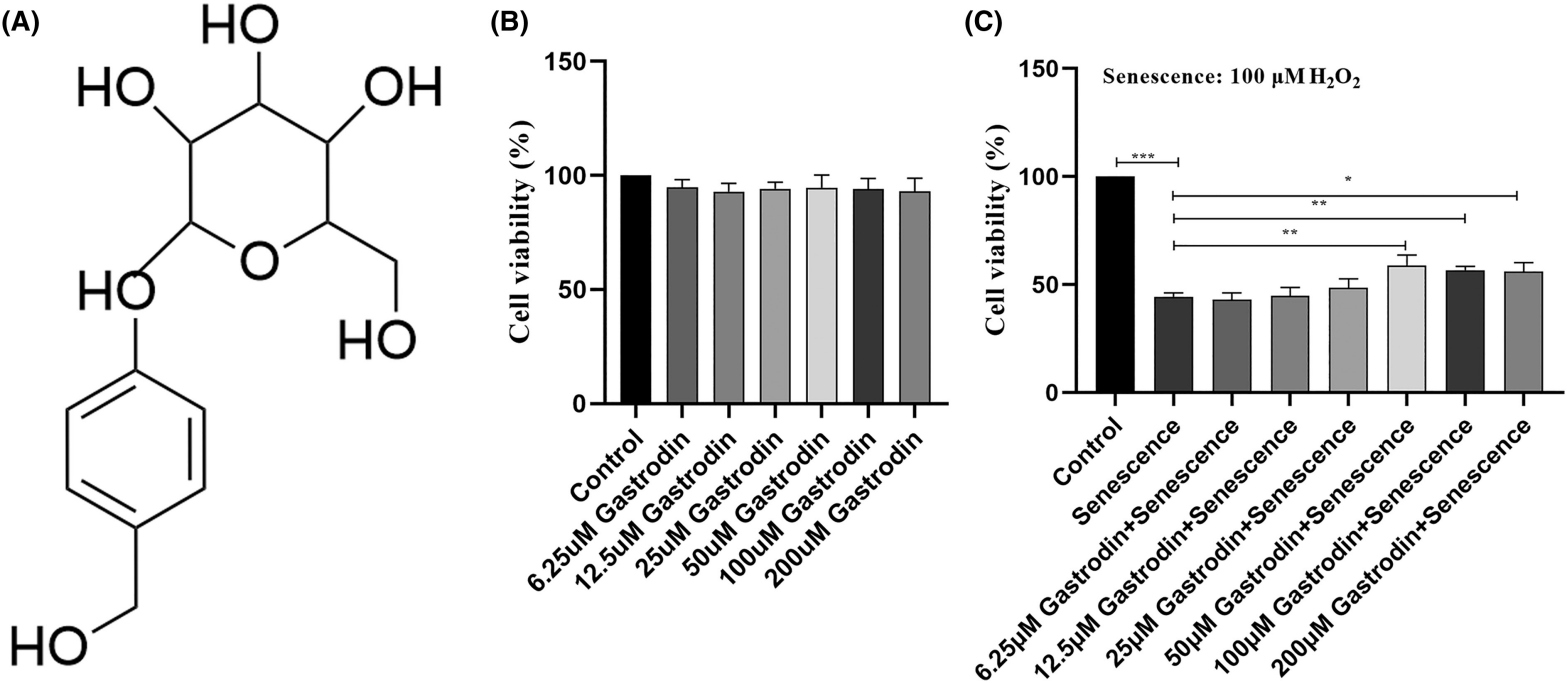
Evaluation of GAS cytotoxicity on HUVECs. (A) Chemical formula of GAS. (B) CCK‐8 assay results on HUVECs exposed to different concentrations of GAS for 24 h. (C) Results of CCK‐8 assays determining the effect of GAS on the viability of H_2_O_2_‐treated HUVECs. *p < 0.05, **p < 0.01, ***p < 0.001 versus control. CCK‐8, Cell Counting Kit‐8; GAS, Gastrodin; HUVECs, human umbilical vein endothelial cell.

Prior to assessing the potential protective effects of GAS (Figure 2A) against H_2_O_2_‐induced senescence in HUVECs, possible cytotoxicity was ruled out by incubating HUVECs with various concentrations of GAS (0, 6.25, 12.5, 25, 50, 100 and 200 μM) for 24 h. CCK‐8 assay results showed that none of the concentrations tested was cytotoxic to HUVECs (Figure 2B). Therefore, HUVECs were pre‐incubated with various GAS concentrations for 6 h, exposed to H_2_O_2_ for 2 h, and further supplemented with GAS (0, 6.25, 12.5, 25, 50, 100 and 200 μM) for an additional 24 h. Results of CCK‐8 assays indicated that GAS significantly improved the viability of senescent HUVECs with the increase of GAS, at concentrations of 50 μM and higher significantly improved the viability of senescent HUVECs (Figure 2C). Therefore, we selected GAS concentrations of 50 and 100 μM to treat cells in the following experiments.

### 3.3 Gastrodin inhibits H_2_O_2_ ‐induced senescence in HUVECs

**FIGURE 3:**
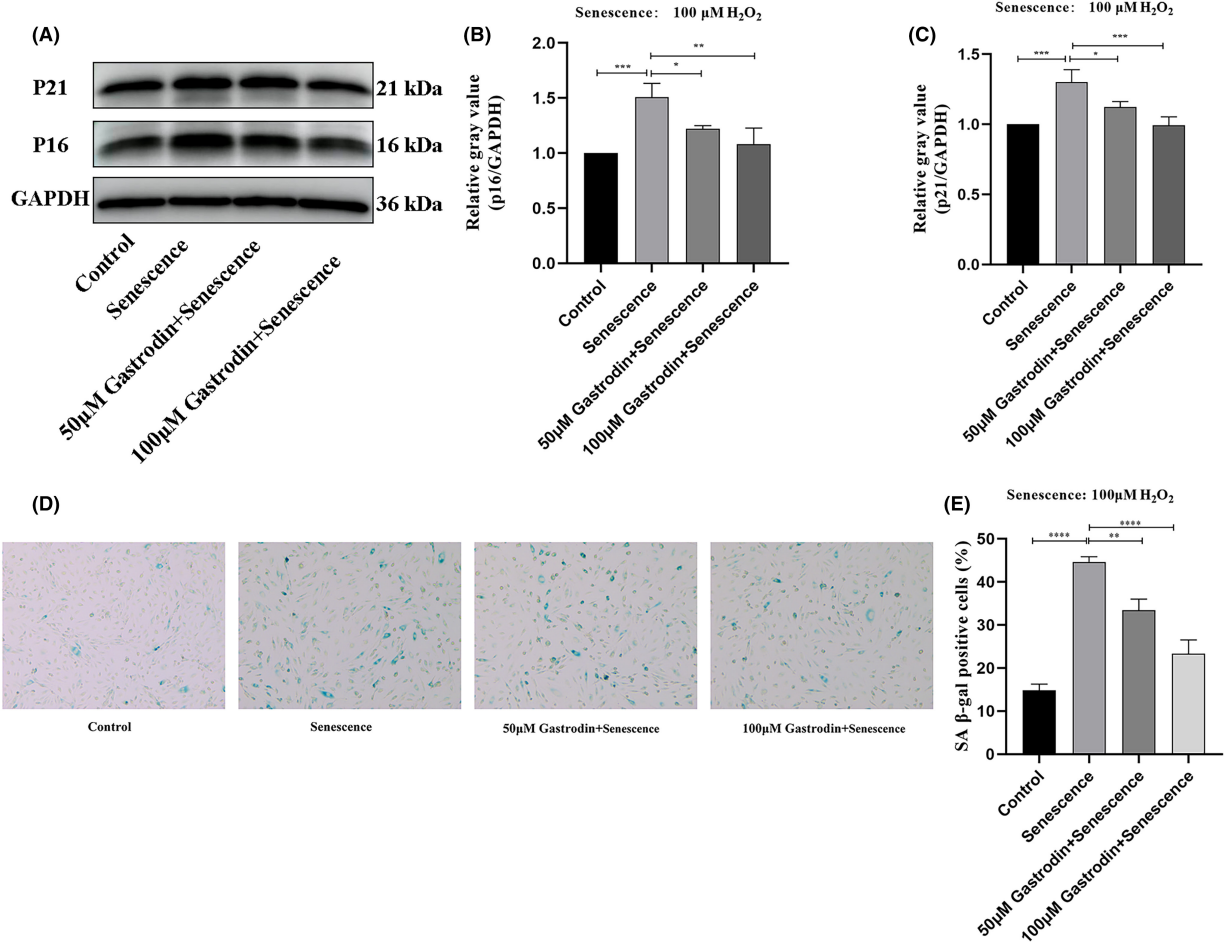
Gastrodin inhibits senescence induced by H_2_O_2_ in HUVECs. (A) Determination of p16 and p21 protein expression by western blotting. (B, C) Densitometric analysis of p16 and p21 protein expression based on western blot assays. (D) Representative images of SA β‐gal staining in cultured HUVECs. Senescent cells become larger and stained blue (E) Quantification of SA β‐gal‐positive cells. (Magnification ×100) *p < 0.05, **p < 0.01 ***p < 0.001 ^****^ *p* < 0.0001. HUVECs, human umbilical vein endothelial cells; SA β‐gal, senescence‐associated β‐galactosidase. Senescence: 100 μM H_2_O_2_.

Next, we evaluated whether the increase in cell viability mediated by GAS in H_2_O_2_‐treated HUVECs was associated with anti‐senescence effects. Western blot analysis showed that in the presence of 50 or 100 μM GAS, after H_2_O_2_ exposure the protein expression of p16 and p21 was significantly decreased. In line with these findings, upon incubation with GAS, the percentage of SA β‐gal‐positive HUVECs was significantly reduced (Figure 3D,E). These results indicate that GAS inhibits H_2_O_2_‐induced senescence in HUVECs.

### 3.4 Gastrodin sustains proliferation and migration in H_2_O_2_ ‐treated HUVECs

**FIGURE 4:**
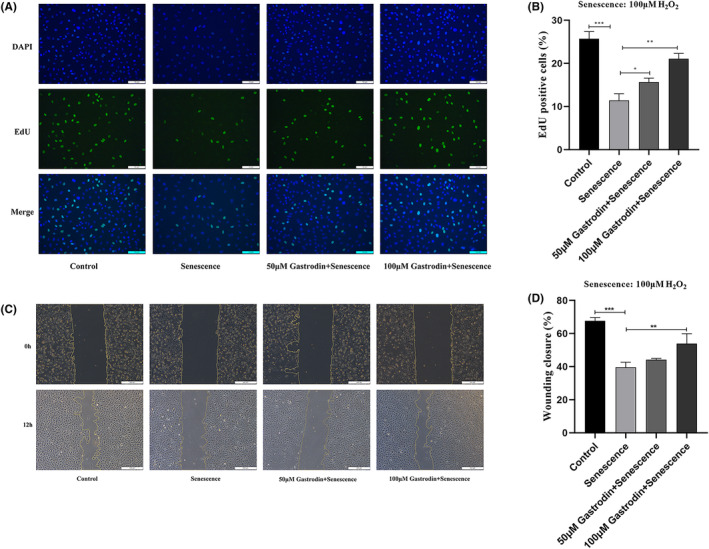
Gastrodin sustains the proliferative and migratory ability of senescent HUVECs. (A) Representative images of EdU straining in cultured HUVECs. (B) Quantification of EdU‐positive cells, indicating active proliferation. (Magnification ×200) (C) Representative images from wound healing assays performed in cultured HUVECs. (Magnification ×50) (D) Quantification of wound closure rates. *p < 0.05, **p < 0.01, ***p < 0.0001. HUVECs, human umbilical vein endothelial cells.

Next, the effects of GAS on the proliferation and migration of senescent HUVECs were evaluated through EdU and wound healing assays, respectively. The results of EdU assays demonstrated that compared to the control group, the rate of proliferation was decreased by H_2_O_2_, and this effect was significantly attenuated after treatment with 50 or 100 μM GAS (Figure 4A,B). In turn, as shown in Figure 4C,D, wound healing assay results demonstrated that cell migration capacity was obviously decreased by H_2_O_2_, and this effect was significantly attenuated in cells co‐incubated with 100 μM GAS. The effect of gastrodin on the migration capacity of ECs was further verified in the Transwell assay. In Figure S1 A,B, it was found that the migration capacity of ECs was significantly reduced in the senescent group compared with the control group. It was further found that gastrodin increased the migration ability of ECs in a concentration‐dependent manner. These results indicated that GAS sustains the proliferative and migratory capacity of vascular ECs during oxidative stress.

### 3.5 Gastrodin counteracts H_2_O_2_ ‐mediated senescence in HUVECs by activating the Nrf2/ HO‐1 antioxidant pathway

**FIGURE 5:**
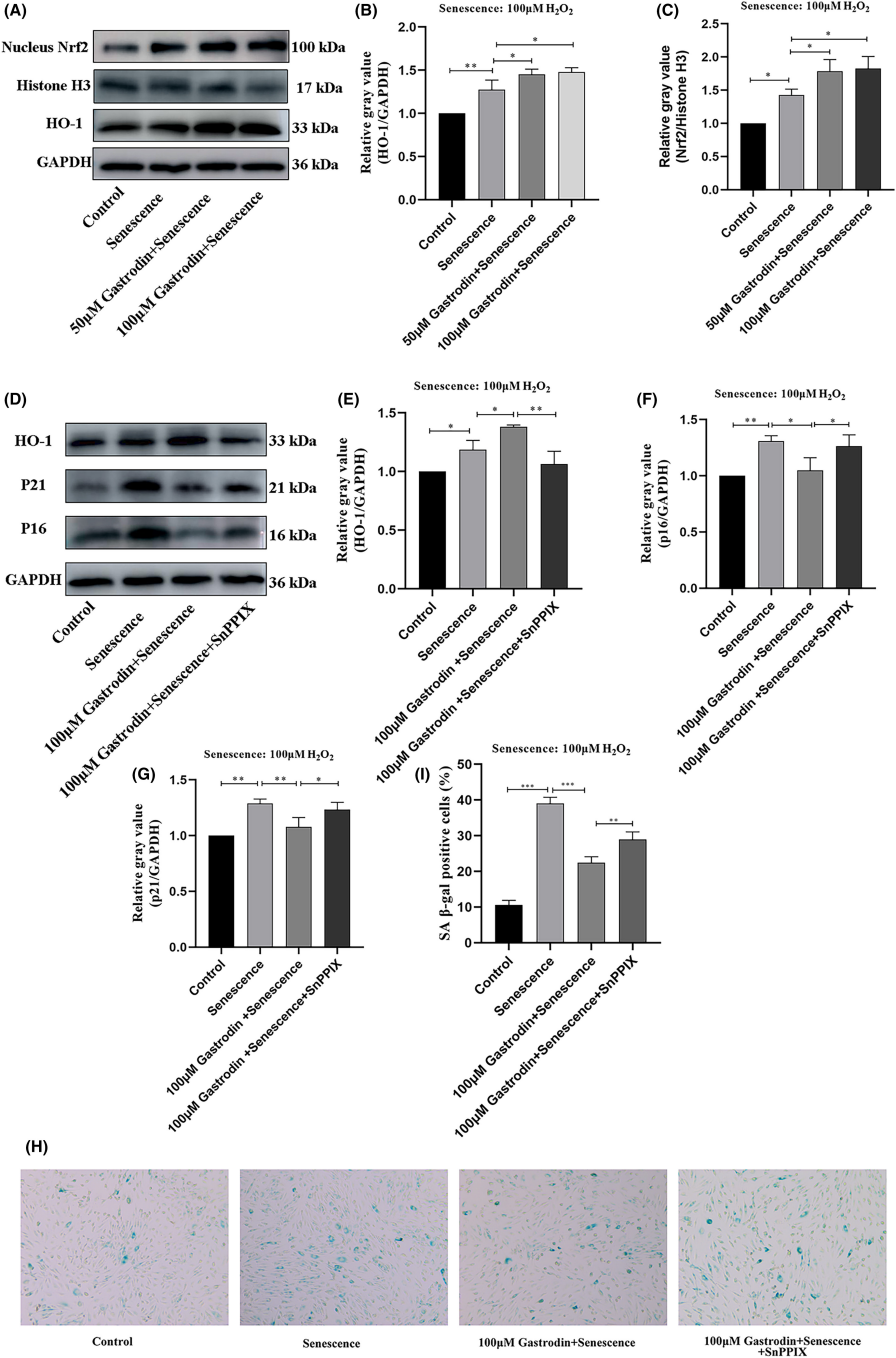
Gastrodin activates Nrf2/HO‐1 signalling in H_2_O_2_‐treated HUVECs. (A) Western blot analysis of Nrf2 and HO‐1 protein expression. (B, C) Densitometric quantification of Nrf2 and HO‐1 protein expression based on western blot data. (D) Analysis of protein levels of HO‐1, p16, and p21 by western blotting. (E–G) Densitometric quantification of HO‐1 (E), p16 (F), and p21 (G) protein expression based on western blot data. (H) Representative images of SA β‐gal staining in cultured HUVECs. Senescent cells become larger and stained blue (Magnification × 100) (I) Quantification of SA β‐gal‐positive cells. *p < 0.05, **p < 0.01, ***p < 0.001. HO‐1, Heme oxygenase‐1; HUVECs, human umbilical vein endothelial cells; Nrf2, Nuclear factor erythroid 2‐related factor 2; SA β‐gal, senescence‐related β‐galactosidase.

Since oxidative stress is the main determinant of H_2_O_2_‐induced cellular senescence, we speculated that the pro‐survival effects of GAS on H_2_O_2_‐exposed HUVECs are mediated by activation of antioxidant responses. To test this hypothesis, we evaluated the effect of GAS on the protein expression of Nrf2, a key regulator of antioxidant gene expression and HO‐1, a main Nrf2 effector. Western blot analyses showed that compared to control cells, the protein expression of both Nrf2 and HO‐1 was increased in HUVECs incubated with H_2_O_2_ alone, and both proteins were further upregulated in cells incubated with H_2_O_2_ in the presence of GAS (Figure 5A–C). To investigate whether GAS‐mediated HO‐1 upregulation influences the expression of senescence markers in H_2_O_2_‐treated HUVECs, SnPPIX, an inhibitor of HO‐1, was applied to HUVEC cultures before H_2_O_2_ exposure. As shown in Figure 5D,E, SnPPIX pre‐treatment abolished the enhancing effect of GAS on HO‐1 expression. Supporting a key role for HO‐1 in GAS‐mediated anti‐senescence effects, additional western blot assays indicated that SnPPIX pre‐treatment negated the suppressive effect of GAS on the expression of the senescence‐related proteins p16 and p21 in H_2_O_2_‐challenged HUVECs (Figure 5F,G).

To further explore whether Nrf2/HO‐1 signalling activation plays an essential role in GAS‐mediated protective effects against HUVEC senescence induced by H_2_O_2_ exposure, we performed SA β‐gal staining in cells pretreated with SnPPIX. As shown in Figure 5H,I, the inhibitory effect of GAS on SA β‐gal expression was abolished after pre‐treatment with SnPPIX. These data indicate that GAS exerts anti‐senescence effects on H_2_O_2_‐treated HUVECs by activating the Nrf2/HO‐1 antioxidant signalling pathway.

### 3.6 Inhibitor blunts gastrodin's proliferative and migratory effects on H_2_O_2_ ‐treated HUVECs

**FIGURE 6:**
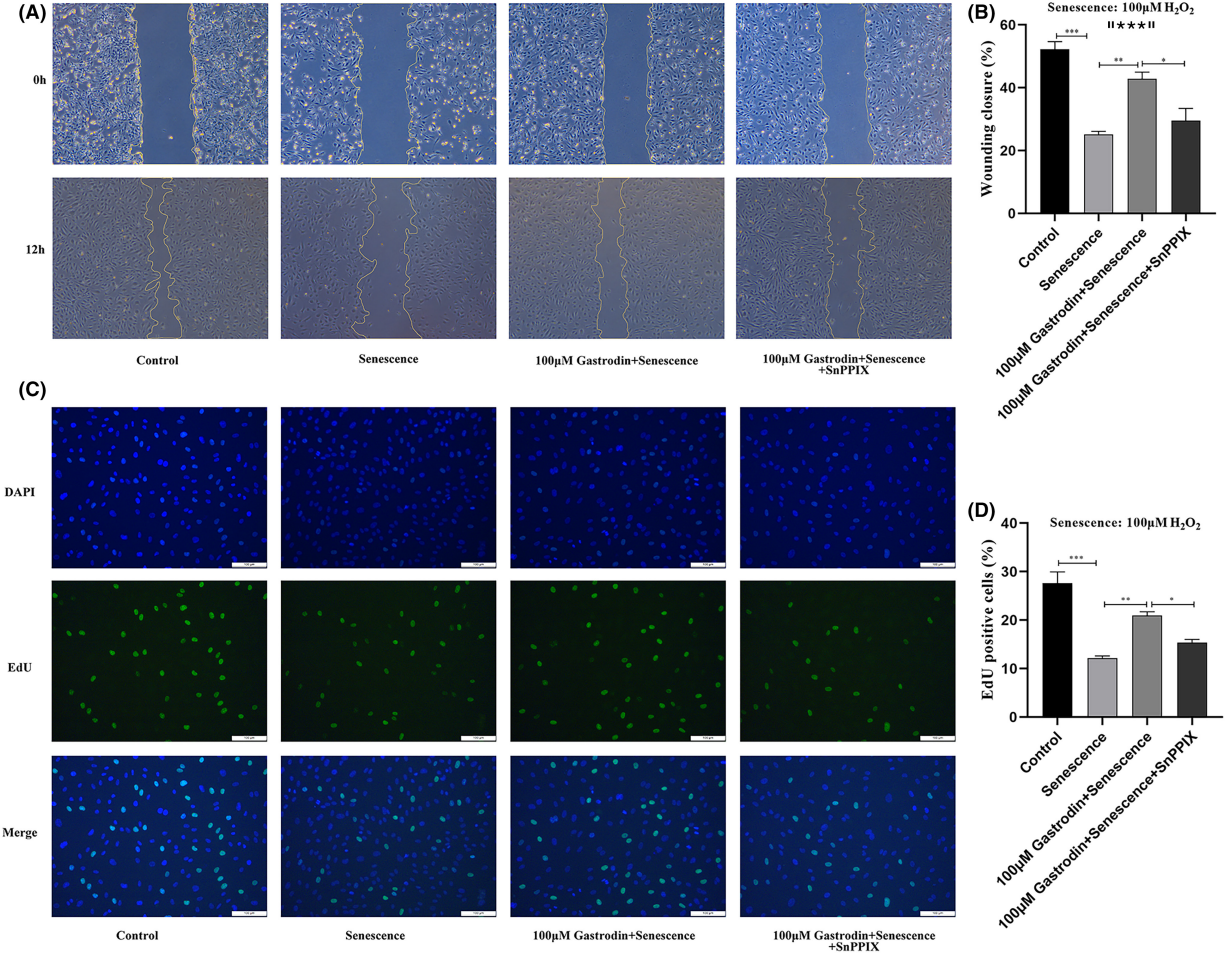
Activation of Nrf2/HO‐1 signalling underlies gastrodin's proliferative and migratory effects on H_2_O_2_‐treated HUVECs. (A) Representative images of EdU staining (cell proliferation assay). (Magnification ×200) (B) Quantification of EdU‐positive cells. (C) Representative images from wound healing assays. (Magnification ×50) (D) Quantification of wound closure rates. *p < 0.01, **p < 0.001, ***p < 0.0001. HO‐1, Heme oxygenase‐1; HUVECs, human umbilical vein endothelial cells; Nrf2, Nuclear factor erythroid 2‐related factor 2.

**FIGURE 7:**
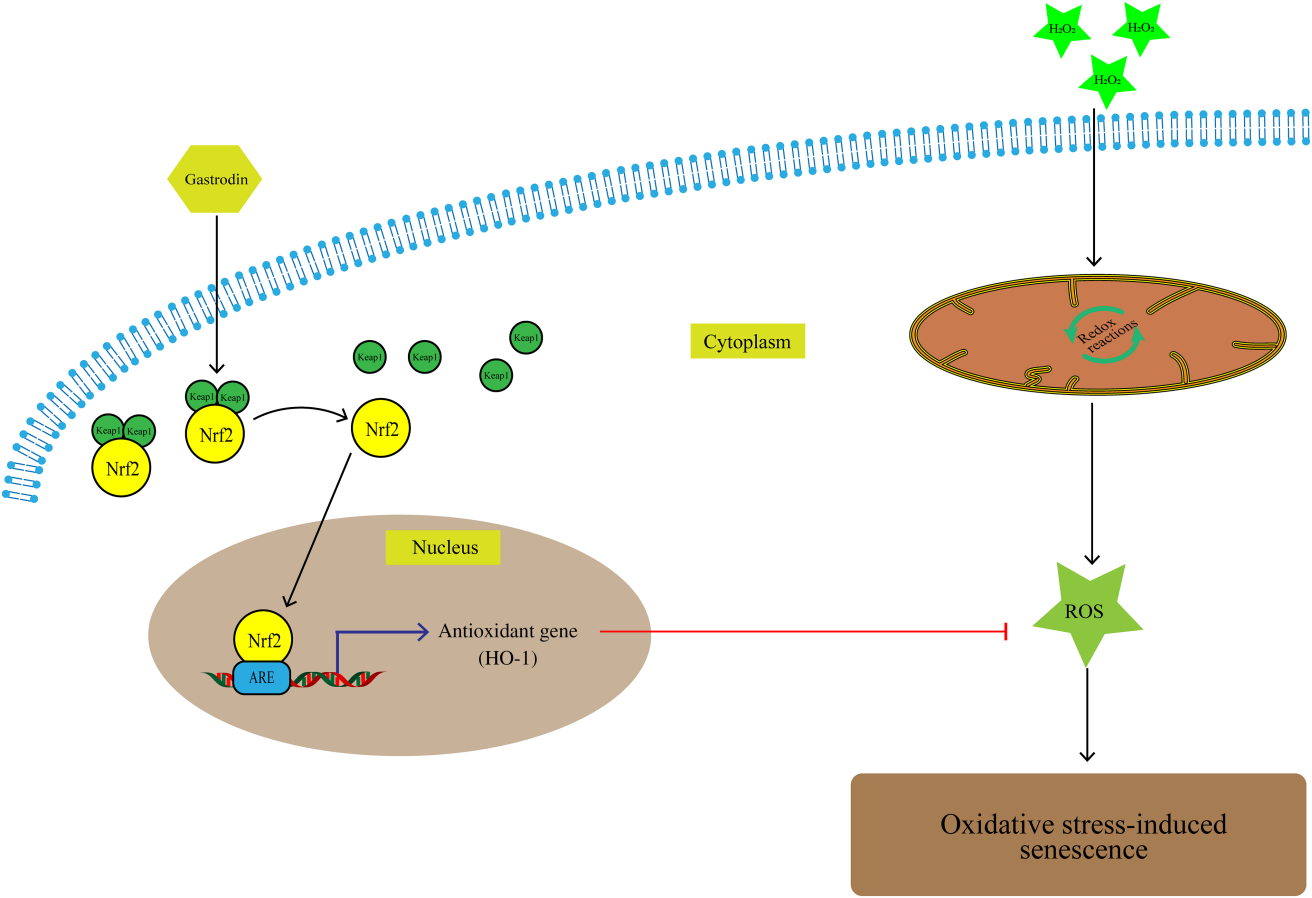
Scheme summarizing the inhibition of H_2_O_2_‐induced HUVECs senescence by gastrodin through the Nrf2/HO‐1 signalling pathway. HUVECs, human umbilical vein endothelial cells; Nrf2, Nuclear factor erythroid 2‐related factor 2; HO‐1, Heme oxygenase‐1; Keap1, Kelch‐like ECH‐associated protein 1 (Keap1); ARE, antioxidant response elements.

Finally, we asked whether GAS‐mediated HO‐1 expression contributes to enhanced proliferative and migratory capacity in HUVECs challenged with H_2_O_2_. As shown in Figure 6A,B, EdU assay results indicated that the proliferative effect of GAS on H_2_O_2_‐treated HUVECs was inhibited when the cells were pre‐incubated with SnPPIX. Similarly, results of wound healing assays showed that SnPPIX pre‐treatment abolished the pro‐migratory effect of GAS on H_2_O_2_‐exposed HUVECs (Figure 6C,D). The effect of gastrodin on EC senescence was also abolished by the HO‐1 inhibitor SnPPIX in the Transwell assay (Figure S1C,D). Collectively, these data imply that the Nrf2/HO‐1 axis plays a major protective role in GAS‐treated, H_2_O_2_‐exposed HUVECs. In conclusion, our study demonstrated that gastrodin alleviates hydrogen peroxide‐induced senescence of human umbilical vein ECs through enhancing the Nrf2/HO‐1 signalling pathway (Figure 7).

## 4 DISCUSSION

In this study, we demonstrated that GAS exposure alleviates oxidative stress‐induced senescence in vascular ECs and enhances their proliferative and migratory potential by stimulating Nrf2/HO‐1 signalling. Two lines of evidence support this conclusion. First, GAS treatment decreased the expression of characteristic senescence markers, namely SA β‐gal, p16 and p21, and enhanced the proliferation and migration ability of H_2_O_2_‐challenged HUVECs. Second, Second, GAS exposure upregulated Nrf2 and HO‐1 protein expression levels in H_2_O_2_‐treated HUVECs, and inhibitor blunted its anti‐senescence, pro‐proliferative and pro‐migratory effects.

Cellular senescence is a multifactorial process, influenced by multiple environmental and genetic factors and characterized by progressive decline in physiological functions, such as growth, proliferation and migration.^28^ Senescent ECs usually present with flattened, enlarged and increasingly polyploid nuclei.^6^, ^29^ Although many endogenous and exogenous factors can promote cellular senescence, they are typically and strongly associated with an imbalance in redox reactions.^30^

Interestingly, an increasing number of studies have found that many TCMs can mitigate cellular senescence. Rutaecarpine, a bioactive alkaloid isolated from Evodia rutaecarpa, delays EC senescence induced by high glucose by regulating the TRPV1/SIRT1 signalling pathway. Astragalus polysaccharides restrain high glucose‐induced senescence and inflammasome activation in rat aortic ECs by modulating the mitochondrial Na+/Ca + exchanger. Tetrahydroxy stilbene glycoside, a major bioactive compound found in *Polygonum multiflorum*, alleviates H_2_O_2_‐induced premature senescence in ECs by modulating the microRNA‐34a/SIRT1 axis. In our study, we present novel evidence for the efficacy of GAS, a TCM herbal extract, in relieving oxidative stress‐induced senescence in vascular ECs via activation of the Nrf2/HO‐1 antioxidant response.

Nrf2, is a member of the Cap ‘n’ collar/basic leucine zipper (CNC‐bZIP) family of transcription factors and acts as a master regulator of cellular redox status in eukaryotic cells and of phase II detoxification responses in mammals.^13^, ^31^ Dose‐ and time‐dependent Nrf2 activation has been detected in a variety of cells upon H_2_O_2_ stimulation.^32^, ^33^ During oxidative stress, the nuclear expression of Nrf2 was increased and activates the transcription of a series of antioxidant genes, including HO‐1, which catalyses the degradation of heme to biliverdin, iron ions and carbon monoxide.^31^ Since this effect is associated with antioxidant, anti‐inflammatory and anti‐apoptotic actions, induction of HO‐1 expression through pharmacological interventions is considered an important therapeutic strategy in vascular diseases.

Consistent with activation of the Nrf2/HO‐1 signalling axis by oxidative stress, we observed that the protein expression of Nrf2 and HO‐1 was upregulated in HUVECs following H_2_O_2_ exposure. However, at the H_2_O_2_ concentration tested (100 mM), the increase in Nrf2 and HO‐1 expression was clearly insufficient to prevent the induction of senescence markers and features in these cells. A previous study demonstrated that GAS can alleviate mouse liver sinusoidal EC damage induced by H_2_O_2_ by stimulating the Nrf2/HO‐1 signalling pathway.^26^ Likewise, Lin et al. showed that following oxidative stress, GAS‐mediated activation of Nrf2/HO‐1 signalling exerts an essential protective role against oxidative damage in HUVECs in vitro and accelerates also wound healing in vivo.^20^ Our results confirmed that GAS activates the Nrf2/HO‐1 pathway in H_2_O_2_‐treated HUVECs and demonstrated also that GAS‐mediated HO‐1 induction is a major determinant of its cytoprotective actions. However, in this work, the effects of GAS on EC senescence were evaluated only in vitro. Thus, further in vivo studies are required to confirm and expand the present findings.

## 5 CONCLUSIONS

Our research showed that GAS counteracts H_2_O_2_‐induced senescence and dysfunction in HUVECs by inhibiting SA β‐gal, p16 and p21 protein expression and by sustaining cell proliferation and migration capacities via enhanced activation of the Nrf2/HO‐1 axis. These findings suggest that GAS may be a valuable therapeutic agent to prevent and treat stroke and other age‐related cerebrovascular diseases.

## AUTHOR CONTRIBUTIONS

**Pengfei Tong:** Conceptualization (equal); data curation (equal); formal analysis (equal); investigation (equal); methodology (equal); resources (equal); software (equal); validation (equal); writing – original draft (equal). **Ke Tian:** Data curation (equal); formal analysis (equal); investigation (equal); methodology (equal). **Jiajia Bi:** Resources (equal); software (equal); validation (equal); visualization (equal). **Ruihua Wang:** Conceptualization (equal); data curation (equal); investigation (equal); supervision (equal); writing – review and editing (equal). **Zhengfeng Wang:** Conceptualization (equal); funding acquisition (equal); supervision (equal); writing – review and editing (equal).

## FUNDING INFORMATION

This work was supported by the Science and Technology Department of Henan Province under Grant [222102310028].

## CONFLICT OF INTEREST STATEMENT

The authors declare that the research was conducted in the absence of any commercial or financial relationships that could be construed as a potential conflict of interest.

## Supporting information


Figure S1.

## Data Availability

The datasets generated in this study are available from the corresponding author upon reasonable request.

## References

[1] Hankey GJ . Stroke. Lancet. 2017;389(10069):641‐654. doi:10.1016/s0140-6736(16)30962-x 27637676

[2] Wei G , Ji X , Bai H , Ding Y . Stroke research in China. Neurol Res. 2006;28(1):11‐15. doi:10.1179/016164106X91807 16464356

[3] Liu H , Chen T , Li N , Wang S , Bu P . Role of SIRT3 in angiotensin II‐induced human umbilical vein endothelial cells dysfunction. BMC Cardiovasc Disord. 2015;15:81. doi:10.1186/s12872-015-0075-4 26223796 PMC4520206

[4] Wang C‐K , Cheng J , Liang X‐G , et al. A H_2_O_2_‐responsive theranostic probe for endothelial injury imaging and protection. Theranostics. 2017;7(15):3803‐3813. doi:10.7150/thno.21068 29109778 PMC5667350

[5] Hafner F , Kieninger A , Meinitzer A , et al. Endothelial dysfunction and brachial intima‐media thickness: long term cardiovascular risk with claudication related to peripheral arterial disease: a prospective analysis. PLoS One. 2014;9(4):e93357. doi:10.1371/journal.pone.0093357 24740106 PMC3989175

[6] Romero A , San Hipólito‐Luengo Á , Villalobos LA , et al. The angiotensin‐(1‐7)/mas receptor axis protects from endothelial cell senescence via klotho and Nrf2 activation. Aging Cell. 2019;18(3):e12913. doi:10.1111/acel.12913 30773786 PMC6516147

[7] Rui YN , Chen Y , Guo Y , et al. Podosome formation impairs endothelial barrier function by sequestering zonula occludens proteins. J Cell Physiol. 2020;235(5):4655‐4666. doi:10.1002/jcp.29343 31637713 PMC7087352

[8] Graves SI , Baker DJ . Implicating endothelial cell senescence to dysfunction in the ageing and diseased brain. Basic Clin Pharmacol Toxicol. 2020;127(2):102‐110. doi:10.1111/bcpt.13403 32162446 PMC7384943

[9] Xiao B , Chai Y , Lv S , et al. Endothelial cell‐derived exosomes protect SH‐SY5Y nerve cells against ischemia/reperfusion injury. Int J Mol Med. 2017;40(4):1201‐1209. doi:10.3892/ijmm.2017.3106 28849073 PMC5593464

[10] Zhang M , Du Y , Lu R , et al. Cholesterol retards senescence in bone marrow mesenchymal stem cells by modulating autophagy and ROS/p53/p21(Cip1/Waf1) pathway. Oxid Med Cell Longev. 2016;2016:7524308. doi:10.1155/2016/7524308 27703600 PMC5040816

[11] Meng J , Lv Z , Qiao X , et al. The decay of redox‐stress response capacity is a substantive characteristic of aging: revising the redox theory of aging. Redox Biol. 2017;11:365‐374. doi:10.1016/j.redox.2016.12.026 28043053 PMC5219648

[12] Davalli P , Mitic T , Caporali A , Lauriola A , D'Arca D . ROS, cell senescence, and novel molecular mechanisms in aging and age‐related diseases. Oxid Med Cell Longev. 2016;2016:3565127. doi:10.1155/2016/3565127 27247702 PMC4877482

[13] Kensler TW , Wakabayashi N , Biswal S . Cell survival responses to environmental stresses via the Keap1‐Nrf2‐ARE pathway. Annu Rev Pharmacol Toxicol. 2007;47:89‐116. doi:10.1146/annurev.pharmtox.46.120604.141046 16968214

[14] Kato K , Logsdon NJ , Shin YJ , et al. Impaired Myofibroblast dedifferentiation contributes to nonresolving fibrosis in aging. Am J Respir Cell Mol Biol. 2020;62(5):633‐644. doi:10.1165/rcmb.2019-0092OC 31962055 PMC7193787

[15] Erusalimsky JD . Vascular endothelial senescence: from mechanisms to pathophysiology. J Appl Physiol (1985). 2009;106(1):326‐332. doi:10.1152/japplphysiol.91353.2008 19036896 PMC2636933

[16] Sies H . Oxidative stress: a concept in redox biology and medicine. Redox Biol. 2015;4:180‐183. doi:10.1016/j.redox.2015.01.002 25588755 PMC4309861

[17] Chang CF , Liu XM , Peyton KJ , Durante W . Heme oxygenase‐1 counteracts contrast media‐induced endothelial cell dysfunction. Biochem Pharmacol. 2014;87(2):303‐311. doi:10.1016/j.bcp.2013.11.002 24239896 PMC3947226

[18] True AL , Olive M , Boehm M , et al. Heme oxygenase‐1 deficiency accelerates formation of arterial thrombosis through oxidative damage to the endothelium, which is rescued by inhaled carbon monoxide. Circ Res. 2007;101(9):893‐901. doi:10.1161/circresaha.107.158998 17885218

[19] Liang R , Zhao Q , Zhu Q , He X , Gao M , Wang Y . Lycium barbarum polysaccharide protects ARPE19 cells against H2O2induced oxidative stress via the Nrf2/HO1 pathway. Mol Med Rep. 2021;24(5):769. doi:10.3892/mmr.2021.12409 34490478 PMC8436232

[20] Lin J , Shi Y , Miao J , et al. Gastrodin alleviates oxidative stress‐induced apoptosis and cellular dysfunction in human umbilical vein endothelial cells via the nuclear factor‐erythroid 2‐related factor 2/Heme Oxygenase‐1 pathway and accelerates wound healing in vivo. Front Pharmacol. 2019;10:1273. doi:10.3389/fphar.2019.01273 31749701 PMC6843024

[21] Tonelli C , Chio IIC , Tuveson DA . Transcriptional regulation by Nrf2. Antioxid Redox Signal. 2018;29(17):1727‐1745. doi:10.1089/ars.2017.7342 28899199 PMC6208165

[22] Wang R , Liu L , Liu H , et al. Reduced NRF2 expression suppresses endothelial progenitor cell function and induces senescence during aging. Aging. 2019;11(17):7021‐7035. doi:10.18632/aging.102234 31494646 PMC6756903

[23] Wang F , Hong Y , Jiang W , et al. ROS‐mediated inflammatory response in liver damage via regulating the Nrf2/HO‐1/NLRP3 pathway in mice with trichloroethylene hypersensitivity syndrome. J Immunotoxicol. 2022;19(1):100‐108. doi:10.1080/1547691x.2022.2111003 36070617

[24] Spatz ES , Wang Y , Beckman AL , et al. Traditional Chinese medicine for acute myocardial infarction in Western medicine hospitals in China. Circ Cardiovasc Qual Outcomes. 2018;11(3):e004190. doi:10.1161/circoutcomes.117.004190 29848478 PMC5882246

[25] Zheng C , Lo CY , Meng Z , et al. Gastrodin inhibits store‐operated Ca^2+^ entry and alleviates cardiac hypertrophy. Front Pharmacol. 2017;8:222. doi:10.3389/fphar.2017.00222 28487655 PMC5404510

[26] Zhang H , Yuan B , Huang H , Qu S , Yang S , Zeng Z . Gastrodin induced HO‐1 and Nrf2 up‐regulation to alleviate H_2_O_2_‐induced oxidative stress in mouse liver sinusoidal endothelial cells through p38 MAPK phosphorylation. Braz J Med Biol Res. 2018;51(10):e7439. doi:10.1590/1414-431X20187439 30156611 PMC6110350

[27] Liao CC , Yu HP , Chou AH , Lee HC , Hu LM , Liu FC . Gastrodin alleviates acetaminophen‐induced liver injury in a mouse model through inhibiting MAPK and enhancing Nrf2 pathways. Inflammation. 2022;45(4):1450‐1462. doi:10.1007/s10753-021-01557-1 35474551

[28] Syslová K , Böhmová A , Mikoška M , Kuzma M , Pelclová D , Kačer P . Multimarker screening of oxidative stress in aging. Oxid Med Cell Longev. 2014;2014:562860. doi:10.1155/2014/562860 25147595 PMC4124763

[29] Jia G , Aroor AR , Jia C , Sowers JR . Endothelial cell senescence in aging‐related vascular dysfunction. Biochim Biophys Acta Mol Basis Dis. 2019;1865(7):1802‐1809. doi:10.1016/j.bbadis.2018.08.008 31109450

[30] Lee JJ , Ng SC , Hsu JY , et al. Galangin reverses H(2)O(2)‐induced dermal fibroblast senescence via SIRT1‐PGC‐1α/Nrf2 signaling. Int J Mol Sci. 2022;23(3):1387. doi:10.3390/ijms23031387 35163314 PMC8836071

[31] Loboda A , Damulewicz M , Pyza E , Jozkowicz A , Dulak J . Role of Nrf2/HO‐1 system in development, oxidative stress response and diseases: an evolutionarily conserved mechanism. Cell Mol Life Sci. 2016;73(17):3221‐3247. doi:10.1007/s00018-016-2223-0 27100828 PMC4967105

[32] Marinho HS , Real C , Cyrne L , Soares H , Antunes F . Hydrogen peroxide sensing, signaling and regulation of transcription factors. Redox Biol. 2014;2:535‐562. doi:10.1016/j.redox.2014.02.006 24634836 PMC3953959

[33] Cheng QQ , Wan YW , Yang WM , et al. Gastrodin protects H9c2 cardiomyocytes against oxidative injury by ameliorating imbalanced mitochondrial dynamics and mitochondrial dysfunction. Acta Pharmacol Sin. 2020;41(10):1314‐1327. doi:10.1038/s41401-020-0382-x 32203078 PMC7608121

