# Supplementary figures and images for: Gastrodin alleviates premature senescence of vascular endothelial cells by enhancing the Nrf2/HO‐1 signalling pathway
Source: J Cell Mol Med. 2023 Dec 25;28(3):e18089. doi: 10.1111/jcmm.18089 (PMC10844697; doi:10.1111/jcmm.18089)

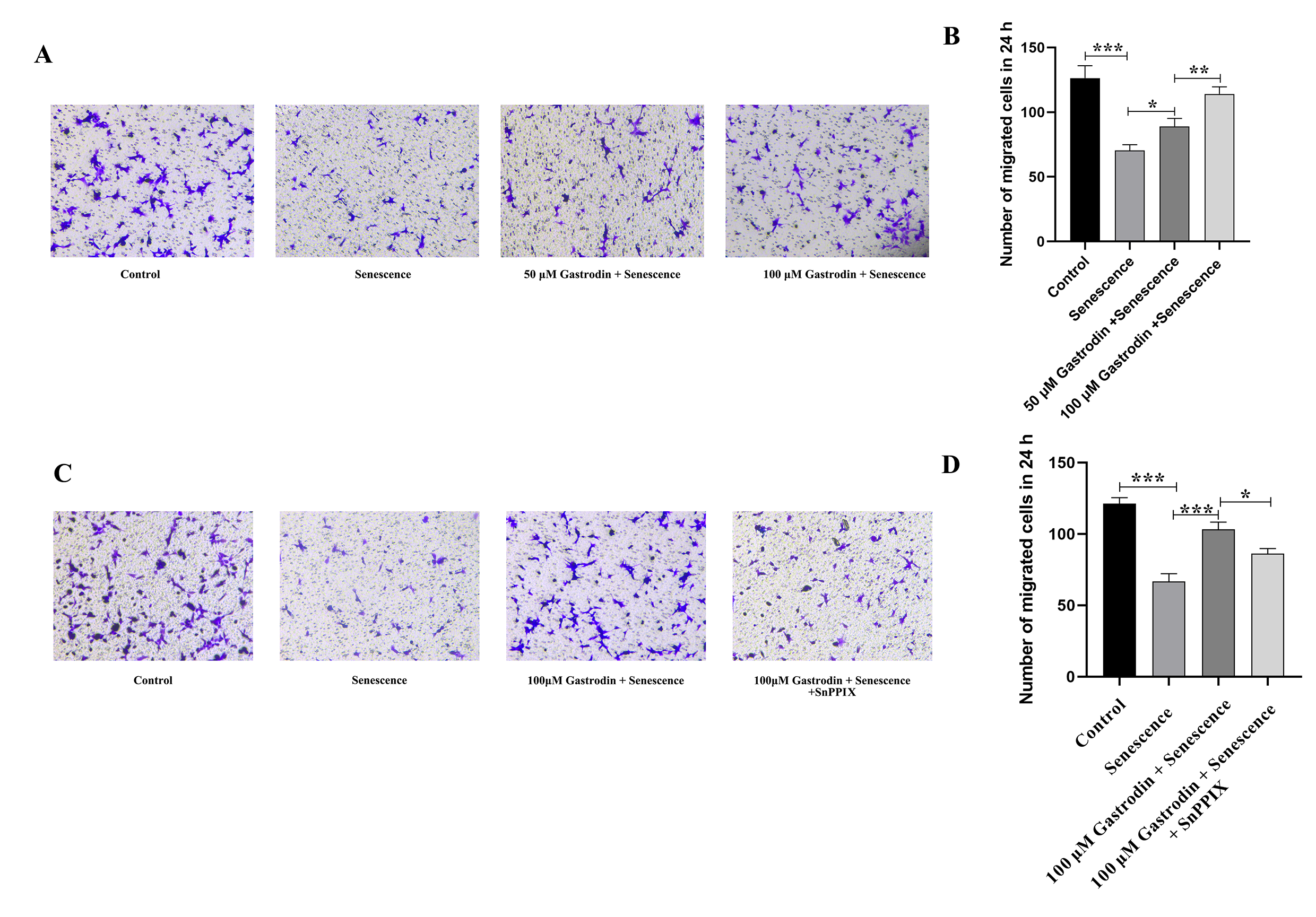

Supplement: Supplementary file 1 — Figure S1. [file JCMM-28-e18089-s001.tif]
